# Metabolomics analysis in adults with high bone mass identifies a relationship between bone resorption and circulating citrate which replicates in the general population

**DOI:** 10.1111/cen.14119

**Published:** 2019-11-20

**Authors:** April Hartley, Lavinia Paternoster, David M. Evans, William D. Fraser, Jonathan Tang, Debbie A. Lawlor, Jon H. Tobias, Celia L. Gregson

**Affiliations:** ^1^ Medical Research Council Integrative Epidemiology Unit Population Health Sciences Bristol Medical School University of Bristol Bristol UK; ^2^ Population Health Sciences Bristol Medical School Bristol University Bristol UK; ^3^ Musculoskeletal Research Unit Translation Health Sciences Bristol Medical School University of Bristol Bristol UK; ^4^ Translational Research Institute The University of Queensland Diamantina Institute Brisbane Qld Australia; ^5^ Department of Medicine Norwich Medical School University of East Anglia Norwich UK; ^6^ National Institute for Health Research Bristol Biomedical Research Centre Bristol UK

**Keywords:** ALSPAC, bone turnover, citrate, high bone mass, metabolomics, triglycerides

## Abstract

**Objective:**

Bone turnover, which regulates bone mass, may exert metabolic consequences, particularly on markers of glucose metabolism and adiposity. To better understand these relationships, we examined cross‐sectional associations between bone turnover markers (BTMs) and metabolic traits in a population with high bone mass (HBM, BMD Z‐score ≥+3.2).

**Design:**

β‐C‐terminal telopeptide of type‐I collagen (β‐CTX), procollagen type‐1 amino‐terminal propeptide (P1NP) and osteocalcin were assessed by electrochemiluminescence immunoassays. Metabolic traits, including lipids and glycolysis‐related metabolites, were measured using nuclear magnetic resonance spectroscopy. Associations of BTMs with metabolic traits were assessed using generalized estimating equation linear regression, accounting for within‐family correlation, adjusting for potential confounders (age, sex, height, weight, menopause, bisphosphonate and oral glucocorticoid use).

**Results:**

A total of 198 adults with HBM had complete data, mean [SD] age 61.6 [13.7] years; 77% were female. Of 23 summary metabolic traits, citrate was positively related to all BTMs: adjusted β_β‐CTX_ = 0.050 (95% CI 0.024, 0.076), *P* = 1.71 × 10^−4^, β_osteocalcin_ = 6.54 × 10^−4^ (1.87 × 10^−4^, 0.001), *P* = .006 and β_P1NP_ = 2.40 × 10^−4^ (6.49 × 10^−5^, 4.14 × 10^−4^), *P* = .007 (β = increase in citrate (mmol/L) per 1 µg/L BTM increase). Inverse relationships of β‐CTX (β = −0.276 [−0.434, −0.118], *P* = 6.03 × 10^−4^) and osteocalcin (−0.004 [−0.007, −0.001], *P* = .020) with triglycerides were also identified. We explored the generalizability of these associations in 3664 perimenopausal women (age 47.9 [4.4] years) from a UK family cohort. We confirmed a positive, albeit lower magnitude, association between β‐CTX and citrate (adjusted β_women_ = 0.020 [0.013, 0.026], *P* = 1.95 × 10^−9^) and an inverse association of similar magnitude between β‐CTX and triglycerides (β = −0.354 [−0.471, −0.237], *P* = 3.03 × 10^−9^).

**Conclusions:**

Bone resorption is positively related to circulating citrate and inversely related to triglycerides. Further studies are justified to determine whether plasma citrate or triglyceride concentrations are altered by factors known to modulate bone resorption, such as bisphosphonates.

## 1 INTRODUCTION

Bone is increasingly recognized to play a role in regulating energy metabolism. Osteocalcin is a measure of osteoblast function and thus bone formation.^1^, ^2^, ^3^ Osteocalcin‐deficient mice have increased blood glucose, reduced insulin levels and an increase in fat mass compared to wild‐type mice.^4^ In human populations, osteocalcin has been inversely associated with fat mass and blood glucose levels.^5^, ^6^

As osteocalcin is an abundant protein in the bone matrix, it can also be used as a marker of bone turnover, the combined process of bone formation and bone resorption.^1^, ^2^ When resorption exceeds formation, age‐related bone loss occurs (potentially leading to osteoporosis).^1^ In clinical practice, bone turnover is commonly measured by N‐terminal propeptide of type 1 procollagen (P1NP, a collagen product of bone formation) and beta collagen type 1 cross‐linked C‐telopeptide (β‐CTX, a collagen product of bone resorption); the latter particularly being used to monitor response to osteoporosis treatments.^1^, ^2^, ^3^ Bone turnover markers (BTMs), which reflect metabolism of type 1 collagen,^1^, ^2^, ^7^ may also aid identification of individuals at risk of fracture.^1^, ^2^

We recently gained understanding of the ‘bone turnover – metabolic phenotype’ by investigating a rare and extreme population with high bone mass (HBM). We previously found HBM to be a sporadic finding of generalized raised bone mineral density (BMD) on dual‐energy X‐ray absorptiometry (DXA) scanning, with a prevalence of 0.18% among a UK DXA‐scanned adult population, characterized by a largely asymptomatic mild skeletal dysplasia.^8^ Compared with relatives with normal BMD, HBM individuals have lower bone turnover, including reduced osteocalcin levels, with increased fat mass in women.^9^

We therefore aimed to understand the relationships between bone turnover and metabolic markers by examining cross‐sectional associations between BTMs and a series of metabolic traits measured using a high‐throughput proton nuclear magnetic resonance spectroscopy (NMR) platform, in HBM individuals. We hypothesized that the predominant associations observed would be between BTMs and metabolic markers of fat metabolism. Furthermore, we aimed to assess the generalizability of any bone turnover‐associated metabolic traits, by examining whether similar relationships exist in unselected individuals of differing age groups from the general population.

## 2 METHODS

### 2.1 High bone mass (HBM) population

The HBM study is a UK‐based multicentred observational study of adults with unexplained HBM. At four of our larger centres, 788 cases of unexplained HBM were identified by screening NHS dual X‐ray absorptiometry (DXA) databases (n = 219 088 DXA images). Full details of DXA database screening and participant recruitment have previously been reported.^8^ In brief, HBM was defined as a) L1 Z‐score of ≥+3.2 plus total hip Z‐score of ≥+1.2 or b) total hip Z‐score ≥+3.2 plus L1 Z‐score of ≥+1.2. Cases with significant osteoarthritis and/or other causes of raised BMD were excluded (eg surgical metalwork, Paget's disease, metastases). Index cases were asked to pass on study invitations to their first‐degree relatives and spouse/partner(s). Relatives/spouses with HBM were in turn asked to pass on study invitations to their first‐degree relatives and spouses. First‐degree relatives and spouses were recruited, in whom HBM status was defined as L1 Z plus total hip Z‐scores of ≥+3.2. Family controls comprised unaffected relatives and spouses (Figure S1). All participants (214 with HBM and 126 family controls without HBM) were clinically assessed by one doctor using a standardized structured history and examination questionnaire, after which nonfasted phlebotomy was performed. Written informed consent was obtained from all participants in line with the Declaration of Helsinki.^10^ Recruitment ran from July 2005 to April 2010. Participants were excluded if they were under 18 years old, pregnant or unable to provide written informed consent for any reason. The study was approved by the Bath Multi‐centre Research Ethics Committee (REC reference 05/Q2001/78) and at each local NHS REC.

### 2.2 Avon longitudinal study of parents and children (ALSPAC)

ALSPAC is a long‐standing prospective cohort study of 14 541 pregnancies with expected delivery dates between 01/04/1991 and 31/12/1992, in Southwest England.^11^, ^12^ Of these pregnancies, 14 676 foetuses resulted in 14 062 live births, with 13 988 children alive at 1 year. When the oldest children were aged approximately 7 years, an attempt was made to augment the initial sample, resulting in 811 additional children being enrolled. We analysed data collected from the mothers (first clinic session December 2008‐July 2011) and offspring when aged 15 years (third clinic). A total of 11 264 (77.5%) mothers were invited, of whom 4832 attended (42.9%). A total of 10 464 (71.2%) offspring were invited, of whom 5506 (52.6%) attended (Figure S2). The study website details all available data through a fully searchable data dictionary: http://www.bristol.ac.uk/alspac/researchers/our-data/. Ethical approval was obtained from the ALSPAC Ethics and Law committee and the local Research Ethics Committees.

### 2.3 Assessment of bone turnover markers

In the HBM population, nonfasted P1NP and total osteocalcin were measured as markers of bone formation and β‐CTX was measured as a marker of bone resorption. In ALSPAC populations, fasted β‐CTX concentration was measured. In all, plasma was separated and frozen within 4 hours to −80°C and BTM concentrations were measured by electrochemiluminescence immunoassays (Roche Diagnostics), with detection limits of 4.0, 0.6 and 0.01 µg/L for P1NP, osteocalcin and β‐CTX, respectively. Reference ranges were supplied by UK Supra Regional Assay Service laboratory (reference range 0.1‐0.5 µg/L for β‐CTX, 20‐110 µg/L for P1NP and 6.8‐32.2 µg/L for osteocalcin). All inter‐ and intra‐assay coefficients of variation were <6%.

### 2.4 Nuclear magnetic resonance (NMR) metabolic profiling

Plasma metabolic profiling was performed using a targeted high‐throughput proton NMR platform, which measures absolute concentrations of over 150 metabolic traits, including 14 lipoprotein subclasses, lipids, glycolysis‐related metabolites, amino acids, ketone bodies and biomarkers of fluid balance and inflammation.^20^, ^22^ The protocol has been published elsewhere.^13^, ^14^, ^15^, ^16^ To reduce the number of statistical tests performed, we specifically focused analyses on the total measures for (nonfasted) lipids, glycerides and phospholipids, apolipoproteins (rather than their subfactions), in addition to all amino acids, ketone bodies, markers of fluid balance and inflammation and low molecular weight metabolites, including glycolysis‐related metabolites. This totalled 23 measures (summarized in Table S1).

### 2.5 Covariates

In the HBM population, researcher‐administered questionnaires quantified bisphosphonate and glucocorticoid use, tobacco and alcohol consumption, menopausal history and use of oestrogen replacement in women. ALSPAC offspring pubertal stage was assessed by Tanner line drawings,^17^, ^18^ using a paper questionnaire sent to all participants prior to clinic attendance. ALSPAC women were asked if they were taking hormone replacement, and to list all current medications, from which bisphosphonate and oral glucocorticoid use was determined. Maternal alcohol consumption was ascertained as part of a postal questionnaire sent in 2010. Women were considered postmenopausal if they had not had a period in the last 12 months or if their periods had stopped due to hysterectomy, ablation/ resection, chemotherapy or other medical reasons.^19^ Height and weight were measured contemporaneous to blood sampling.

### 2.6 Statistical analysis

Histograms of exposure and outcome variables were visually inspected to identify skewed variables. Descriptive statistics were summarized as mean with standard deviations (SD) (or median [interquartile range, IQR] for skewed variables) and counts (percentages). Associations between BTMs and metabolic traits were assessed by multivariable linear regression, with standardized variables to allow comparisons between metabolic traits. Robust standard errors (SEs), which remain unbiased if data are skewed, and confidence intervals (CIs) were estimated. Repeating all analyses log‐transforming outcome variables did not alter our findings, and therefore, original units with robust SEs are presented.

To account for intrafamily clustering, associations between BTMs and metabolic traits were determined using generalized estimating equation (GEE) linear regression. Analyses were initially performed unadjusted (model 1), then adjusted for the a priori confounders age and sex (model 2) and finally also adjusted for additional confounders height, weight, menopausal status, bisphosphonate and glucocorticoid use (model 3). All BTMs were then added to model 3 (model 4). We tested for interaction between β‐CTX concentration and HBM status using model 3. Due to the number of tests performed in our initial metabolite screen (23 outcomes), we adjusted our *P* value threshold of significance to account for multiple testing (α threshold 0.05/23 = 0.002).

Analyses of ALSPAC populations also used standard multivariable linear regression with robust SEs. For the mother's cohort, model 3 was adjusted for age, height, weight, menopausal status and fasting time prior to sample collection (<8 or ≥8 hours). As only 14 mothers reported bisphosphonate use and 12 oral glucocorticoid use, we removed these mothers in a sensitivity analysis. For the offspring population, model 3 was adjusted for age, sex, height, weight, Tanner stage and time of sample collection (AM or PM). All adjusted analyses, including in the HBM population, were performed with the metabolic traits in their original units to allow comparison between populations. All analyses were performed in Stata version 13 (Statacorp), and figures were generated using R version 3.5.1.

## 3 RESULTS

### 3.1 Characteristics of the HBM population

The 198 HBM individuals had mean (SD) age 62 (14) years, BMI 30.5 (5.8) kg/m^2^, and 77% were female. Median (IQR) BTM concentrations were as follows: β‐CTX 0.17 (0.12‐0.25) µg/L, P1NP 32.0 (23.0‐44.0) µg/L and osteocalcin 16.6 (13.1‐21.2) µg/L (Table S2).

### 3.2 Unadjusted analysis of metabolic traits and bone turnover in individuals with HBM

**Figure 1:**
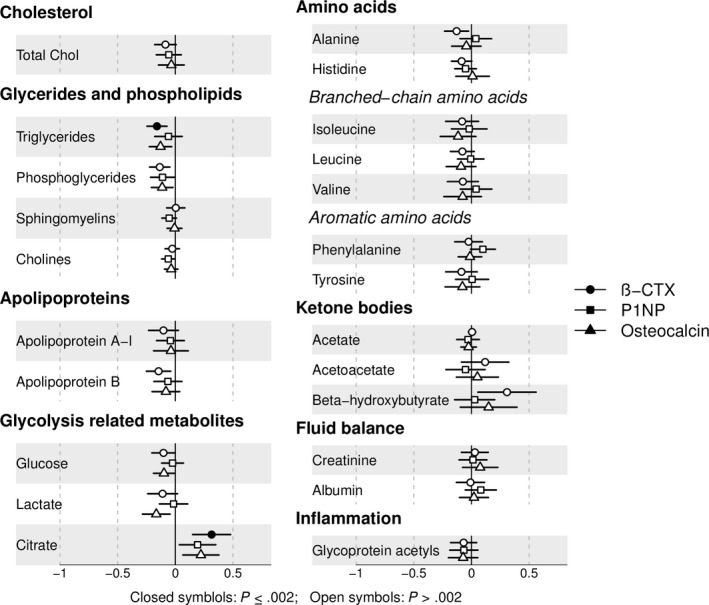
Unadjusted associations between bone turnover markers and metabolic traits for all individuals with HBM. Points represent the SD increase in metabolic trait per SD increase in bone turnover marker. Horizontal lines represent 95% confidence intervals. Results are presented in SD units for comparison between metabolic traits. N ranges from 186 to 198 depending on metabolite. Abbreviations: β‐CTX: collagen type 1 cross‐linked C‐telopeptide; P1NP: N‐terminal propeptide of type 1 procollagen. Total C: total cholesterol. [Colour figure can be viewed at https://www.wileyonlinelibrary.com]

**Figure 2:**
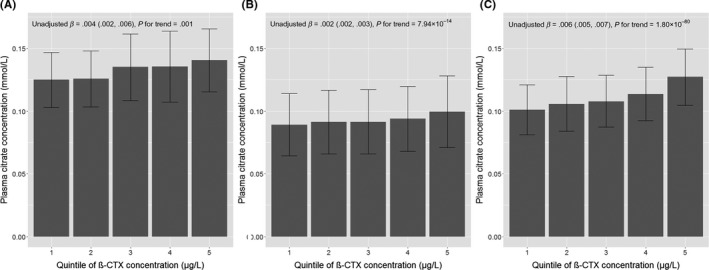
Mean citrate concentrations by quintiles of β‐CTX in the (A) high bone mass, (B) ALSPAC mothers and (C) ALSPAC offspring populations. β represents the increase in citrate in mmol/L per increase in β‐CTX quintile from unadjusted analyses. Abbreviations: β‐CTX, collagen type 1 cross‐linked C‐telopeptide

Of 23 metabolic traits, plasma citrate was positively related to all three BTMs (β_β‐CTX_ = 0.31 [0.15, 0.48], *P* = 1.89 × 10^−4^, β_P1NP_ = 0.19 [0.03, 0.35], *P* = .017 and β_Osteocalcin_ = 0.22 [0.07, 0.38], *P* = .006, β represents the SD increase in citrate per SD increase in BTM) (Figure 1), but only the β‐CTX‐citrate association met our corrected *P* value threshold (Table 1 shows results where β represents the mmol/L increase in citrate per 1 µg/L increase in BTM). Mean (SD) citrate concentration was 0.13 (0.03) mmol/L and increased by quintile of β‐CTX (Figure 2A). Both β‐CTX and osteocalcin were inversely associated with triglycerides (standardized β = −0.16 [−0.25, −0.07], *P* = 3.32 × 10^−4^, β = −0.13 [−0.23, −0.03], *P* = .009 respectively), whilst P1NP was not. Nominal inverse associations between all three BTMs and phosphoglycerides, P1NP and cholines, β‐CTX and apolipoprotein B, β‐CTX/ osteocalcin and glucose, osteocalcin and lactate, β‐CTX and alanine, with a positive association between β‐CTX and β‐hydroxybutyrate, were detected (0.002 < *P* ≤ .05).

**Table 1:** Multivariable associations between bone turnover markers and citrate/triglycerides in individuals with HBM

N = 198	Model 1		Model 2		Model 3		Model 4	
Exposure	β (95% CI)	*P* value	β (95% CI)	*P* value	β (95% CI)	*P* value	β (95% CI)	*P* value
Citrate								
β‐CTX	0.055 (0.026, 0.083)	1.89 × 10^−4^	0.048 (0.022, 0.075)	2.95 × 10^−4^	0.050 (0.024, 0.076)	1.71 × 10^−4^	0.054 (0.009, 0.098)	.019
P1NP	2.36 × 10^−4^ (4.21 × 10^−5^, 4.30 × 10^−4^)	.017	2.35 × 10^−4^ (5.13 × 10^−5^, 4.18 × 10^−4^)	.012	2.40 × 10^−4^ (6.49 × 10^−5^, 4.14 × 10^−4^)	.007	−3.60 × 10^−5^ (−2.88 × 10^−4^, 2.16 × 10^−4^)	.779
Osteocalcin	6.90 × 10^−4^ (2.03 × 10^−4^, 0.001)	.006	6.61 × 10^−4^ (1.76 × 10^−4^, 0.001)	.008	6.54 × 10^−4^ (1.87 × 10^−4^, 0.001)	.006	1.84 × 10^−6^ (−6.98 × 10^−4^, 7.01 × 10^−4^)	.996
Triglycerides								
β‐CTX	−0.288 (−0.445, −0.131)	3.32 × 10^−4^	−0.298 (−0.465, −0.130)	5.03 × 10^−4^	−0.276 (−0.434, −0.118)	6.03 × 10^−4^	−0.377 (−0.628, −0.125)	.003
P1NP	−0.001 (−0.002, 0.001)	.327	−0.001 (−0.002, 0.001)	.332	−0.001 (−0.002, 0.001)	.277	−0.002 (−0.013, 0.034)	.516
Osteocalcin	−0.004 (−0.007, −0.001)	.009	−0.004 (−0.007, −0.001)	.010	−0.004 (−0.007, −0.001)	.020	0.002 (−3.27 × 10^−4^, 0.004)	.104

Note

β represents the increase in citrate/triglycerides in mmol/L per 1 µg/L increase in bone turnover marker. Model 1: unadjusted; Model 2: adjusted for age and sex; Model 3: adjusted for age, sex, height, weight, menopause, bisphosphonate and oral glucocorticoid use; Model 4: Adjusted as per model 3 plus other bone turnover markers.

Abbreviations: β‐CTX, collagen type 1 cross‐linked C‐telopeptide; P1NP, N‐terminal propeptide of type 1 procollagen.

### 3.3 Adjusted analysis of metabolic traits and bone turnover in individuals with HBM

As citrate was most strongly related to bone turnover, it was prioritized for further analysis. The associations between citrate and all three BTMs were unchanged by adjustment for confounders (Table 1). When combining all three BTMs in model 4, only β‐CTX remained independently associated with citrate, with similar effect sizes as seen before adjustment (β_model 1_ = 0.06 [0.03, 0.08], *P* = 1.89 × 10^−4^ and β_model 4_ = 0.05 [0.01, 0.10], *P* = .019, β represents the unit increase in citrate in mmol/L per 1 µg/L increase in β‐CTX).

β‐CTX‐triglyceride and osteocalcin‐triglyceride associations were also robust to covariate adjustment (Table 1). β‐CTX was inversely associated with all triglyceride subvariables (triglycerides in VLDL, LDL and HDL), particularly VLDL triglycerides (Table S3). The association between osteocalcin and triglycerides appeared driven by VLDL. As seen with citrate, when combining all BTMs in the same model, only β‐CTX was independently associated with total, VLDL and LDL triglycerides, and osteocalcin was independently associated with HDL triglycerides (Table 1, Table S3). Triglycerides were not related to citrate (age‐ and sex‐adjusted standardized β = −0.038 [−0.191, 0.114]).

### 3.4 Generalizability of the association between β‐CTX and metabolic traits

We aimed to assess whether bone resorption is similarly associated with citrate in different populations: perimenopausal women with normal BMD (mean [SD] total hip T‐score + 0.24 [1.6]) and adolescents from the ALSPAC cohorts, in whom citrate and β‐CTX (but not P1NP or osteocalcin) had been contemporaneously measured. Of 3664 mothers with mean age 47.9 (4.4) years, 77% were premenopausal. Median β‐CTX and mean citrate concentrations were 0.25 (0.18‐0.35) µg/L and 0.09 (0.03) mmol/L, respectively. Of 2492 adolescents, with mean age 15.4 (0.3) years, 53% were female. Median β‐CTX and the mean citrate concentrations were 0.94 (0.66‐1.39) µg/L and 0.11 (0.02) mmol/L, respectively (Table S4).

Among ALSPAC mothers, a strong positive association was seen between β‐CTX and citrate (mean citrate concentrations increased by quintile of β‐CTX; Figure 2B), robust to confounder adjustment (model 3; Table 2). The magnitude of the relationship was less marked than that seen in the HBM population (fully adjusted β_HBM_ = 0.050 [0.024, 0.076] vs β_mothers_ = 0.020 [0.013, 0.026], β = mmol/L increase in citrate per 1 µg/L increase in β‐CTX). The association between β‐CTX and triglycerides also replicated in ALSPAC mothers (Table 2).

**Table 2:** associations between β‐CTX and citrate and triglycerides in the ALSPAC maternal and adolescent populations

	Model 1			Model 2			Model 3		
	β	95% CI	*P* value	β	95% CI	*P* value	β	95% CI	*P* value
Maternal population N = 3664									
Citrate	0.026	0.020, 0.032	1.28 × 10^−16^	0.022	0.016, 0.028	5.10 × 10^−12^	0.020	0.013, 0.026	1.95 × 10^−9^
Total TGs	−0.414	−0.530, −0.298	3.31 × 10^−12^	−0.502	−0.624, −0.380	1.17 × 10^−15^	−0.354	−0.471, −0.237	3.03 × 10^−9^
VLDL TGs	−0.356	−0.453, −0.259	8.33 × 10^−13^	−0.409	−0.512, −0.307	7.28 × 10^−15^	−0.274	−0.372, −0.176	4.00 × 10^−8^
LDL TGs	−0.017	−0.030, −0.005	.008	−0.035	−0.049, −0.022	1.95 × 10^−7^	−0.030	−0.043, −0.016	1.60 × 10^−5^
HDL TGs	−0.026	−0.034, −0.018	3.43 × 10^−11^	−0.035	−0.043, −0.027	1.76 × 10^−17^	−0.032	−0.040, −0.024	4.86 × 10^−14^
Adolescent population N = 2492									
Citrate	0.018	0.016, 0.019	4.39 × 10^−106^	0.023	0.021, 0.025	1.06 × 10^−93^	0.022	0.020, 0.024	2.10 × 10^−74^
Total TGs	−0.024	−0.047, −0.002	.034	−0.010	−0.043, 0.022	.535	0.041	0.007, 0.076	.019
VLDL TGs	−0.001	−0.020, 0.018	.919	−0.021	−0.050, 0.007	.141	0.028	−0.002, 0.058	.068
LDL TGs	−0.012	−0.015, −0.009	3.61 × 10^−13^	0.007	0.003, 0.011	3.16 × 10^−4^	0.007	0.003, 0.012	.001
HDL TGs	−0.005	−0.006, −0.003	7.48 × 10^−11^	0.001	−0.001, 0.003	.465	0.002	2.93 × 10^−4^, 0.005	.026

Note

β represents the change in citrate or triglycerides in mmol/L per 1 µg/L increase in β‐CTX. Model 1: unadjusted; Model 2: adjusted for age; Model 3: adjusted for age, height, weight, menopause, <8 h of fasting in the maternal population and age, sex, height, weight, Tanner stage and time of sample collection in the adolescent population.

In adolescents, β‐CTX quintile was strongly related to citrate (Figure 2C). In adjusted analyses (model 3), a 1 µg/L increase in β‐CTX was associated with a 0.022 (0.020, 0.024) mmol/L increase in citrate, *P* = 2.10 × 10^−74^ (Table 2). The magnitude of the association between β‐CTX and citrate in adolescents was less than that seen in the HBM population. Inverse associations between β‐CTX and total, LDL and HDL triglycerides were also observed in the adolescent population (Table 2), but with a much smaller effect size than seen in the adult populations. After full adjustment (model 3), β‐CTX remained positively related to total, LDL and HDL triglycerides. Triglycerides were inversely related to citrate in both ALSPAC populations (fully adjusted β_mothers_ = −0.059 [−0.090, −0.028] and β_adolescents_ = −0.095 [−0.135, −0.055], β represents the SD increase in triglycerides per SD increase in citrate). However, adjustment for triglycerides did not attenuate the relationship between β‐CTX and citrate in either population.

### 3.5 Associations between β‐CTX and citrate by HBM status

**Figure 3:**
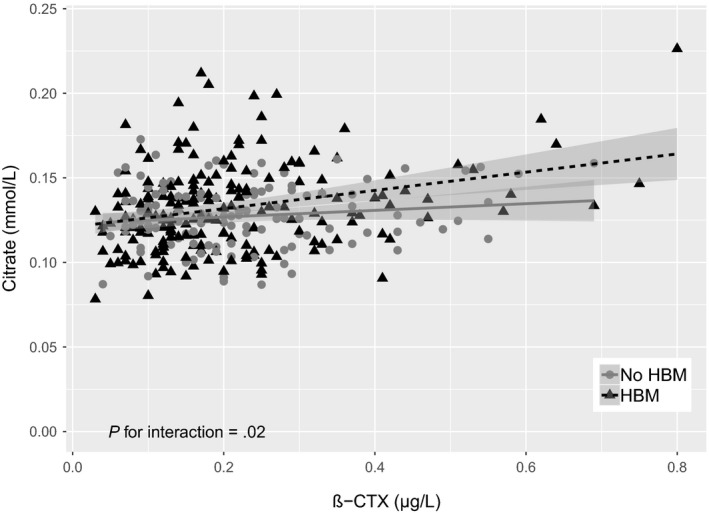
Associations between β‐CTX and citrate in individuals with HBM and family members with normal BMD. N_HBM_ = 198 and N_Relatives_ = 122. Regression lines represent the unadjusted associations between β‐CTX and citrate (β_HBM_ = 0.055 [0.026, 0.083] and β_No HBM_ = 0.022 [−0.003, 0.046] where β represents the increase in mmol/L of citrate per 1 µg/L increase in β‐CTX. After adjusting for age, sex, height, weight, menopause, bisphosphonate use and oral glucocorticoid use (model 3), β‐CTX was still associated with citrate in individuals with HBM (0.050 [95% CI 0.024, 0.076], *P* = 1.71 × 10^−4^) but no association was seen in relatives with normal BMD (8.03 × 10^−4^ [−0.024, 0.026], *P* = .95)

Whilst strong associations were observed between β‐CTX and citrate in all three study populations (HBM individuals, ALSPAC mothers, ALSPAC children), beta coefficients were greater in HBM individuals. To confirm that relationships between β‐CTX and citrate are relatively strong in HBM cases, we tested for an interaction between β‐CTX concentration and citrate according to HBM status, by combining individuals with HBM with their family controls with normal BMD (n = 122, Figure S1). These family controls were younger than the HBM individuals (mean 55.0 vs 61.6 years), less commonly female (44% vs 77%), taller (mean 171.7 vs 166.9 cm) and had lower BMD (mean TH‐BMD Z‐score 0.53 vs 3.02). Median β‐CTX and mean citrate concentrations in the family controls were 0.20 (0.11, 0.28) and 0.13 (0.03) mmol/L, respectively. There was strong evidence for a lower age and sex‐adjusted mean β‐CTX concentration in HBM cases compared to their relatives and weak evidence for a higher mean citrate concentration (mean differences = −0.047 [−0.078, −0.016] µg/L and 0.005 [−2.76 × 10^−4^, 0.010] mmol/L for β‐CTX and citrate, respectively). After adjustment (model 3), no association was seen between β‐CTX and citrate in these family controls; however, the sample size was small (n = 122) and confidence intervals wide (β = 0.002[−0.02, 0.03], *P* = .9). A likelihood ratio test confirmed a difference in the association between β‐CTX and citrate according to HBM status (*P* = .02; Figure 3).

### 3.6 Sensitivity analyses

Adjusting for alcohol and creatinine levels in all populations and excluding ALSPAC mothers reporting bisphosphonates or glucocorticoid use did not change conclusions. The association between β‐CTX and citrate was similar in pre‐ and postmenopausal ALSPAC mothers (*P* for interaction = .3), and between those fasting <8 vs ≥8 hours before sample collection (*P* for interaction = .8).

## 4 DISCUSSION

We report a positive association between β‐CTX and plasma citrate, and consistent but weaker associations between osteocalcin/ P1NP and citrate. Furthermore, β‐CTX and osteocalcin both demonstrated inverse associations with plasma triglycerides in individuals with unexplained HBM, despite adjustment for a range of confounders. Associations between the bone resorption marker, β‐CTX, and citrate and total plasma triglycerides were independent of the two bone formation markers, osteocalcin and P1NP. This positive association between β‐CTX and citrate was further observed in perimenopausal women and adolescents from the ALSPAC population‐based cohort.

Citrate is synthesized in mitochondria from acetyl‐CoA and oxaloacetate during the Krebs cycle, where most remains, regulating energy production.^20^, ^21^ Hence, soft tissue cellular metabolism is not considered a major source of plasma citrate.^21^ Approximately 80% of citrate is stored in bone and 2% of bone content is citrate.^22^ Citrate, found between hydrated layers of bone mineral and which binds to the surface of apatite crystals, is thought to prevent formation of larger crystals and thereby maintain bone structural properties.^23^, ^24^ Human osteoblasts can produce citrate; it is hypothesized that citrate is incorporated into bone directly from osteoblast secretion, and that, as bone is resorbed and both bone collagen and mineral are degraded, citrate is released into the circulation generating the major source of plasma citrate.^25^ This concurs with the positive relationships we observed between citrate and both age and bone resorption and an inverse association recently identified between β‐CTX and citrate in a smaller sample from the ALSPAC adolescent population.^26^ Due to its suggested association with bone mineral, we hypothesize that plasma citrate may provide information on turnover of bone mineral during bone resorption.

Stronger citrate‐β‐CTX associations in the context of HBM compared with individuals with normal BMD may simply reflect the greater quantity of bone in the HBM skeleton and therefore a greater source of citrate. We have previously shown that HBM individuals have increased cortical volumetric BMD measured by pQCT, possibly due to reduced bone turnover allowing more time for secondary mineralization.^27^ Alternatively, the mineral platelets may be structured differently in HBM, contributing to increased bone strength,^27^ which may result in citrate being released more readily during bone resorption.

The inverse association between β‐CTX and triglycerides in the adult HBM and perimenopausal populations is consistent with previous findings from the European Male Ageing Study (EMAS); mean β‐CTX concentrations were lower in male individuals with serum triglyceride concentrations above 150 mg/dL, independent of other components of the metabolic syndrome such as hyperglycaemia.^28^ As we observed, increased osteocalcin has also been associated with reduced triglycerides in adults.^28^, ^30^ The metabolic impact of osteocalcin has further been demonstrated in animal studies, where osteocalcin‐deficient mice display a distinct metabolic phenotype with greater accumulation of fat mass and higher serum triglyceride levels.^4^ In our analyses, the inverse association between osteocalcin and triglycerides was not independent of β‐CTX. It is important to note that we determined associations between *total* osteocalcin and serum triglycerides, rather than the *uncarboxylated* form proposed to be metabolically active.^4^ Yet, our finding that β‐CTX, rather than osteocalcin, was independently associated with triglycerides raises the possibility that β‐CTX influences triglycerides via a separate pathway from osteocalcin. As this analysis is cross‐sectional, we are unable to determine whether increased β‐CTX causes decreased triglyceride levels or vice versa, yet a recent analysis did not find evidence of a causal pathway between triglycerides and BMD after accounting for pleiotropy, consistent with the lack of any causal effect of triglycerides on bone turnover.^31^

In adolescents, we observed the opposite direction of effect between β‐CTX and triglycerides, after adjustment for covariates: β‐CTX was positively related to triglycerides after adjustment for weight. One possible explanation is that during adolescence, increased bone resorption likely reflects bone modelling during growth rather than bone remodelling, as indicated by the positive association between β‐CTX and periosteal circumference previously reported in this adolescent population.^32^ Pubertal growth may increase both bone modelling and fat storage concurrently, with higher associated plasma triglyceride levels. Whilst in mature adults, bone remodelling predominates, and hence, the direction of association reverses.

### 4.1 Strengths and limitations

Strengths include the unique HBM population plus the ability to evaluate generalizability of findings in large population‐based cohorts of perimenopausal women and adolescents. All cohorts had detailed phenotypic data which allowed for models to be adjusted for a range of potential confounders. The metabolomics platform used is highly reproducible and allows efficient quantification of a larger number of biomarkers at scale.^33^

Nevertheless, this cross‐sectional study is unable to examine directions of causality. HBM study samples had been stored at −80°C for up to 10 years before metabolomics analysis; however, previous studies suggest that long‐term storage is unlikely to significantly affect citrate measurements.^34^ The effect of different storage conditions and freeze‐thaw cycles on metabolic trait concentrations may affect lipids, alanine and glucose 35; reassuringly our association between β‐CTX and plasma triglycerides replicated with a similar effect size in the ALSPAC maternal cohort. Citrate has established dietary sources which may explain the clear positive association with fasting duration. β‐CTX is also affected by fasting time, with β‐CTX levels increasing with fasting.^36^ A weaker association was observed between β‐CTX and citrate in those ALSPAC mothers with shorter fasts. The samples collected from the HBM population were not collected when fasting. As the HBM population is predominantly female, we wanted to replicate our analysis in a female population. The ALSPAC maternal cohort is predominantly perimenopausal compared to the HBM population which is mainly postmenopausal; however, as far as we are aware, the ALSPAC maternal population is the largest available cohort of women with measured β‐CTX and citrate. Finally, our study provides limited data as to how β‐CTX relates to citrate and triglycerides in adult men, or adults with low bone mass, in whom further analyses are required.

## 5 CONCLUSIONS

We have identified that plasma citrate is positively associated with β‐CTX in two separate adult populations. Given that citrate binds to apatite nanocrystals,^23^ we hypothesize that circulating citrate may reflect breakdown of bone mineral. Further studies are justified to explore whether plasma citrate concentration is altered by factors known to modulate bone resorption, such as bisphosphonates, to determine the direction of causality.

## CONFLICT OF INTEREST

DAL has received support in the last 10 years from the UK Medical Research Council, National Institute of Health Research, British Heart Foundation, Diabetes UK, Wellcome Trust, the European Research Council, US National Institute of Health and from Roche Diagnostics and Medtronic Ltd for research unrelated to that presented here. WDF has received consultancy fees from Siemens, Becton Dickinson and Roche.

## Data Availability

The HBM data that support the findings of this study are available on request from the corresponding author. The data are not publicly available due to privacy or ethical restrictions. ALSPAC data access is through a system of managed open access. The ALSPAC access policy details how data can be accessed by researchers: http://www.bristol.ac.uk/media-library/sites/alspac/documents/researchers/data-access/ALSPAC_Access_Policy.pdf.
