# Supplementary material for: Metabolomics analysis in adults with high bone mass identifies a relationship between bone resorption and circulating citrate which replicates in the general population
Source: Clin Endocrinol (Oxf). 2019 Nov 20;92(1):29–37. doi: 10.1111/cen.14119 (PMC7017780; doi:10.1111/cen.14119)
Supplement: Supplementary file 1 [file CEN-92-29-s001.docx]

**Metabolomics analysis in adults with High Bone Mass identifies a relationship between bone resorption and circulating citrate which replicates in the general population**

**Supplementary Material**

**April Hartley, Lavinia Paternoster, David M Evans, William D Fraser, Jonathan Tang, Debbie A Lawlor, Jon H Tobias, Celia L Gregson**

**Supplementary Tables and Figures**

**Supplementary Figure S1: Flowchart detailing derivation of study sample in the HBM population and the relative/spouse population with normal BMD**

**
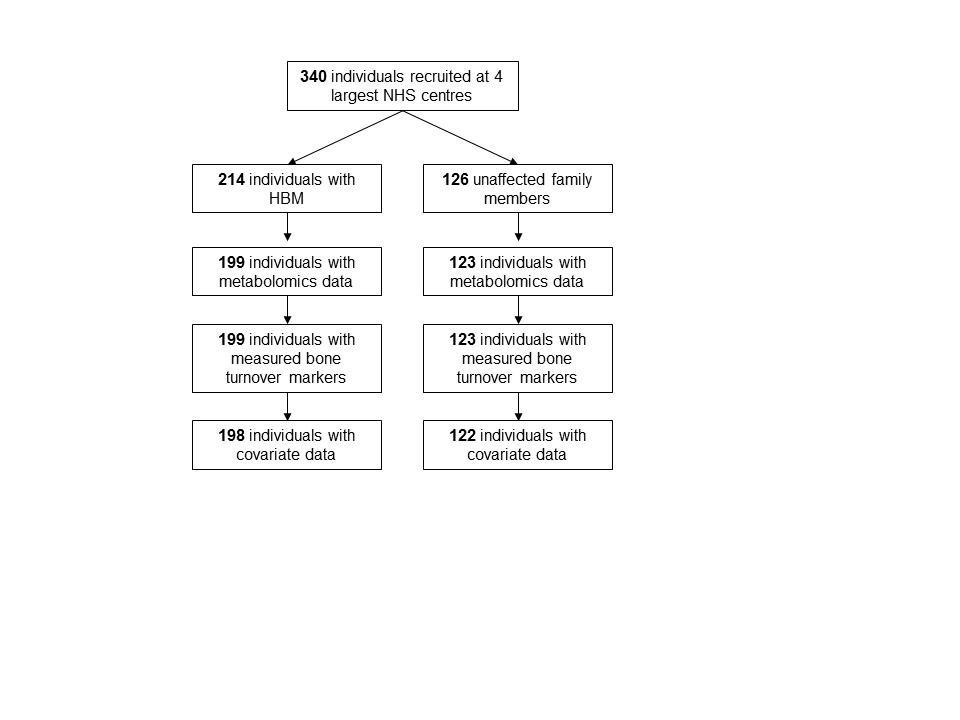
**

**Supplementary Figure S2: Flowchart detailing derivation of study sample in the ALSPAC (A) maternal and (B) offspring cohorts**

**(A) (B)**

**
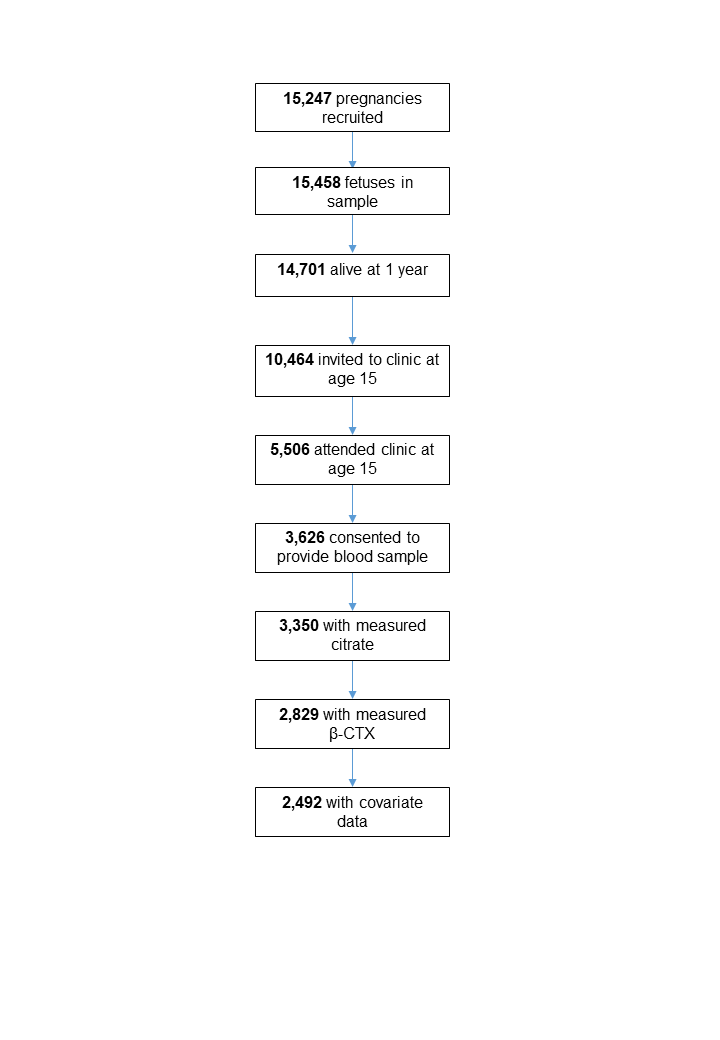
**
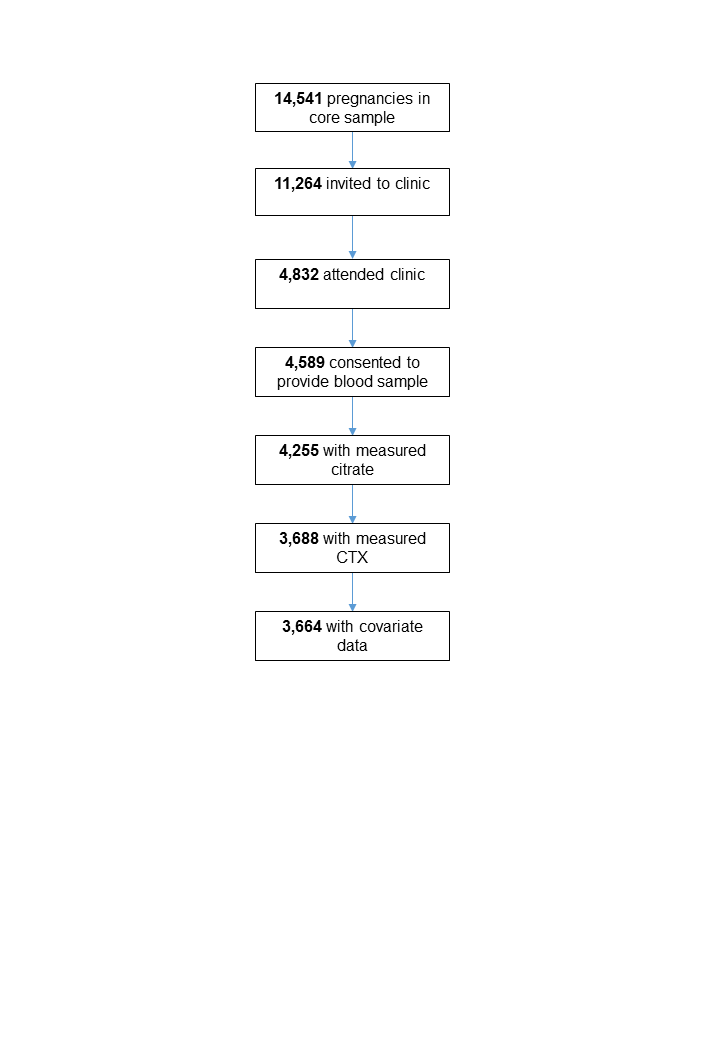


Abbreviations: β-CTX: serum collagen type 1 cross-linked C-telopeptide

**Supplementary Table S1: Summary statistics for the 23 metabolic traits included in our initial analysis in the HBM population**

| **Metabolic trait** | | **N** | **Mean (SD)** | **Median (IQR)** |
| --- | --- | --- | --- | --- |
| **Cholesterol** | | | | |
|  | *Total cholesterol* | 198 | 0.600 (0.299) | 0.504 (0.407, 0.724) |
| **Glycerides and phospholipids** | | | | |
|  | *Total triglycerides* | 198 | 0.672 (0.210) | 0.635 (0.518, 0.766) |
|  | *Phosphoglycerides* | 198 | 0.331 (0.149) | 0.294 (0.239, 0.384) |
|  | *Sphingomyelins* | 198 | 0.202 (0.057) | 0.190 (0.172, 0.216) |
|  | *Cholines* | 198 | 0.659 (0.190) | 0.615 (0.538, 0.710) |
| **Apolipoproteins** | | | | |
|  | *Apolipoprotein A1* | 198 | 0.540 (0.048) | 0.535 (0.506, 0.570) |
|  | *Apolipoprotein B* | 198 | 0.298 (0.090) | 0.281 (0.239, 0.334) |
| **Glycolysis related metabolites** | | | | |
|  | *Glucose* | 197 | 3.736 (1.247) | 3.444 (3.099, 3.862) |
|  | *Lactate* | 197 | 1.200 (0.472) | 1.094 (0.942, 1.416) |
|  | *Citrate* | 198 | 0.132 (0.025) | 0.129 (0.114, 0.146) |
| **Amino acids** | | | | |
|  | *Alanine* | 198 | 0.308 (0.054) | 0.304 (0.271, 0.345) |
|  | *Histidine* | 198 | 0.025 (0.010) | 0.024 (0.020, 0.028) |
|  | *Isoleucine* | 198 | 0.044 (0.015) | 0.042 (0.034, 0.052) |
|  | *Leucine* | 198 | 0.066 (0.019) | 0.063 (0.053, 0.073) |
|  | *Valine* | 198 | 0.138 (0.038) | 0.134 (0.111, 0.159) |
|  | *Phenylalanine* | 186 | 0.061 (0.012) | 0.060 (0.055, 0.066) |
|  | *Tyrosine* | 198 | 0.051 (0.012) | 0.049 (0.042, 0.058) |
| **Ketone bodies** | | | | |
|  | *Acetate* | 197 | 0.123 (0.089) | 0.115 (0.102, 0.128) |
|  | *Acetoacetate* | 197 | 0.012 (0.010) | 0.009 (0.007, 0.013) |
|  | *Beta-hydroxybutyrate* | 198 | 0.152 (0.071) | 0.131 (0.112, 0.167) |
| **Fluid balance** | | | | |
|  | *Creatinine* | 198 | 0.060 (0.013) | 0.058 (0.051, 0.065) |
|  | *Albumin* | 198 | 0.078 (0.007) | 0.078 (0.075, 0.082) |
| **Inflammation** | | | | |
|  | *Glycoprotein acetyls* | 198 | 1.319 (0.210) | 1.308 (1.178, 1.446) |

All metabolic traits measured in mmol/L except for apolipoproteins which are measured in units of g/L

**Supplementary Table S2: Descriptive characteristics of the HBM population and their unaffected relatives and spouses with normal BMD**

|  | **HBM individuals**  **(N=198)** | **Family members with normal BMD (N=122)** | ***p* value for difference** |
| --- | --- | --- | --- |
|  | **Mean (SD)** | **Mean (SD)** |  |
| Age (years) | 61.6 (13.7) | 55.0 (16.2) | 1.26x10^-4^ |
| Height (cm) | 166.9 (8.9) | 171.7 (10.5) | 1.45x10^-5^ |
| Weight (kg) | 84.9 (16.8) | 84.0 (17.4) | 0.664 |
| BMI (kg/m^2^) | 30.5 (5.8) | 28.4 (4.9) | 0.001 |
| L1 BMD^b^ | 1.40 (0.16) | 1.08 (0.16) | 1.34x10^-50^ |
| L1 BMD Z-score^c^ | 3.85 (1.34) | 0.36 (1.25) | 5.36x10^-69^ |
| Max TH BMD^d^ | 1.25 (0.18) | 0.99 (0.14) | 3.13x10^-34^ |
| Max TH BMD Z-score^e^ | 3.02 (1.16) | 0.53 (0.89) | 1.55x10^-57^ |
| Physical Activity  (MET-minutes/ week)^f^ | 3479.0 (3293.0) | 4155.7 (5760.0) | 0.804 |
|  | **Median (IQR)** | **Median (IQR)** |  |
| β-CTX (μg/L)^a^ | 0.17 (0.12, 0.25) | 0.20 (0.11, 0.28) | 0.214 |
| P1NP (μg/L)^a^ | 32.0 (23.0, 44.0) | 34.0 (26.0, 44.0) | 0.339 |
| Osteocalcin (μg/L)^a^ | 16.6 (13.1, 21.2) | 18.0 (14.6, 23.0) | 0.007 |
|  | **N (%)** | **N (%)** |  |
| Gender (Female) | 152 (76.8) | 54 (44.3) | 3.69x10^-9^ |
| Postmenopausal^g^ | 130 (86) | 29 (54) | 1.70x10^-6^ |
| History of bisphosphonate use | 8 (4.0) | 2 (1.6) | 0.231 |
| History of oral glucocorticoid use | 48 (24.2) | 18 (14.8) | 0.042 |
| Prior/current smoking | 114 (57.6) | 58 (47.5) | 0.080 |
| Alcohol Consumption |  |  | 0.002 |
| *None* | 57 (28.8) | 21 (17.2) |  |
| *Occasional* | 22 (11.1) | 10 (8.2) |  |
| *Regular* | 99 (50.0) | 61 (50.0) |  |
| *Heavy* | 20 (10.1) | 30 (24.6) |  |

^a^*p* value represents difference in log-transformed concentrations due to skewed distributions; ^b^N=318: 121 controls, 197 cases; ^c^N=314: 195 cases, 119 controls; ^d^N=315: 195 cases, 120 controls; ^e^N=312: 193 cases, 119 controls; ^f^ N=264: 162 cases, 102 controls; ^g^individuals currently going through menopause included in this category

Max TH BMD is the highest recorded BMD from the left and right hips. Total weekly MET-minutes is calculated as the energy requirement of each activity type multiplied by duration and summed across all three activity types (27). History of oral glucocorticoid use is based on self-report of current or past oral glucocorticoid use for any duration.

Abbreviations: BMI: body mass index; β-CTX: collagen type 1 cross-linked C-telopeptide; P1NP: N-terminal propeptide of type 1 procollagen; BMD: Bone Mineral Density; TH: total hip.

**Supplementary Table S3: associations between bone turnover markers and triglycerides and triglyceride subclass variables in individuals with HBM**

|  |  | **Model 1** | | **Model 2** | | **Model 3** | | **Model 4** | |
| --- | --- | --- | --- | --- | --- | --- | --- | --- | --- |
|  |  | **β (95% CI)** | ***p* value** | **β (95% CI)** | ***p* value** | **β (95% CI)** | ***p* value** | **β (95% CI)** | ***p* value** |
| **β-CTX** | **VLDL TGs** | -0.239  (-0.375, -0.102) | 6.11x10^-4^ | -0.247  (-0.392, -0.101) | 9.05x10^-4^ | -0.228  (-0.365, -0.090) | 0.001 | -0.308  (-0.529, -0.086) | 0.006 |
|  | **LDL TGs** | -0.025  (-0.041, -0.009) | 0.002 | -0.026  (-0.043, -0.009) | 0.002 | -0.025  (-0.041, -0.009) | 0.003 | -0.044  (-0.068, -0.020) | 3.69x10^-4^ |
|  | **HDL TGs** | -0.009  (-0.016, -0.002) | 0.013 | -0.009  (-0.016, -0.002) | 0.015 | -0.008  (-0.015, -0.001) | 0.027 | -0.005  (-0.017, 0.007) | 0.444 |
| **Osteocalcin** | **VLDL TGs** | -0.003  (-0.006, -0.001) | 0.014 | -0.003  (-0.006, -0.001) | 0.015 | -0.003  (-0.006, -3.17x10^-4^) | 0.028 | -0.001  (-0.006, 0.003) | 0.600 |
|  | **LDL TGs** | -2.65x10^-4^  (-0.001, 1.45x10^-4^) | 0.205 | -2.71x10^-4^  (-0.001, 1.43x10^-4^) | 0.199 | -2.40x10^-4^  (-0.001, 1.75x10^-4^) | 0.257 | 2.07x10^-6^  (-6.64x10^-4^, 6.68x10^-4)^ | 0.995 |
|  | **HDL TGs** | -2.21x10^-4^  (-3.65x10^-4^, -7.83x10^-5^) | 0.002 | -2.18x10^-4^  (-3.57x10^-4^, -7.79x10^-5^) | 0.002 | -1.86x10^-4^  (-3.20x10^-4^, -5.25x10^-5^) | 0.006 | -2.58x10^-4^  (-4.87x10^-4^, -2.81x10^-5^) | 0.028 |
| **P1NP** | **VLDL TGs** | -0.001  (-0.002, 0.001) | 0.324 | -0.001  (-0.002, 0.001) | 0.330 | -0.001  (-0.002, 0.001) | 0.270 | 0.001  (-4.71x10^-4^, 0.003) | 0.157 |
|  | **LDL TGs** | -3.73x10^-5^  (-1.89x10^-4^, 1.15x10^-4^) | 0.631 | -3.78x10^-5^  (-1.90x10^-4^, 1.15x10^-4^) | 0.627 | -3.83x10^-5^  (-1.84x10^-4^, 1.08x10^-4^) | 0.608 | 1.79x10^-4^  (-2.84x10^-5^, 3.87x10^-4^) | 0.091 |
|  | **HDL TGs** | -2.77x10^-5^  (-9.40x10^-5^, 3.87x10^-5^) | 0.414 | -2.60x10^-5^  (-9.12x10^-5^, 3.93x10^-5^) | 0.435 | -2.80x10^-5^  (-8.84x10^-5^, 3.24x10^-5^) | 0.364 | 7.07x10^-5^  (-2.54x10^-5^, 1.67x10^-4^) | 0.149 |

β represents the increase in triglycerides in mmol/L per 1μg/L increase in bone turnover marker.

Model 1: unadjusted; Model 2: adjusted for age and sex; Model 3: adjusted for age, sex, height, weight, menopause, bisphosphonate and oral glucocorticoid use; Model 4: Adjusted as per model 3 plus other bone turnover markers

Abbreviations: β-CTX: collagen type 1 cross-linked C-telopeptide; P1NP: N-terminal propeptide of type 1 procollagen; TGs: Triglycerides.

**Supplementary Table S4: Descriptive characteristics of the ALSPAC populations included in these analyses**

|  | **Maternal** | **Adolescent** |
| --- | --- | --- |
|  | **N=3,664** | **N=2,492** |
|  | **Mean (SD)** | |
| Age (years) | 47.9 (4.4) | 15.4 (0.3) |
| Height (cm) | 164.1 (6.1) | 169.6 (8.4) |
| Weight (kg) | 71.2 (14.2) | 61.9 (11.5) |
| BMI (kg/m^2^) | 26.4 (5.1) | 21.5 (3.4) |
| TBFM (kg)^a^ | 26.2 (9.7) |  |
| TBLM (kg)^a^ | 41.0 (4.7) |  |
| TH BMD (g cm^-2^)^b^ | 1.03 (0.14) |  |
| TH BMC (g)^b^ | 32.1 (5.1) |  |
| TBLH BMD^c^ |  | 1.035 (0.088) |
| Plasma citrate (mmol/L) | 0.09 (0.03) | 0.11 (0.02) |
|  | **Median (IQR)** | |
| β-CTX (μg/L) | 0.25 (0.18, 0.35) | 0.94 (0.66, 1.39) |
| Total triglycerides (mmol/L) | 0.92 (0.71, 1.23) | 0.82 (0.69, 1.02) |
| Triglycerides in VLDL (mmol/L) | 0.51 (0.35, 0.77) | 0.51 (0.40, 0.67) |
| Triglycerides in LDL (mmol/L) | 0.16 (0.13, 0.19) | 0.12 (0.10, 0.14) |
| Triglycerides in HDL (mmol/L) | 0.13 (0.11, 0.15) | 0.10 (0.09, 0.11) |
|  | **N (%)** | |
| Gender (Female) | 3,664 (100.0) | 1,308 (52.5) |
| Postmenopausal | 849 (23.2) |  |
| Estrogen replacement use | 158 (4.3) |  |
| Tanner Stage^d^ |  |  |
| *<Stage 5* |  | 1,261 (50.6) |
| *Stage 5* |  | 1,231 (49.4) |
| Bisphosphonate use | 14 (0.4) |  |
| Oral glucocorticoid use | 12 (0.3) |  |
| Fasted <8 hours | 673 (18.4) |  |
| Time of sample collection (AM) |  | 1,404 (56.3) |
| Alcohol consumption^e^ |  |  |
| *Less than once weekly* | 1,052 (43.0) | 1,607 (76.0) |
| *At least once weekly* | 1,394 (57.0) | 509 (24.0) |

^a^N=3,554, ^b^N=3,555, ^d^N=2,457, ^e^N (maternal)=2,446 and N (adolescent)=2,116

^d^Tanner stage below 5 represents an adolescent still going through puberty

Abbreviations: β-CTX: serum collagen type 1 cross-linked C-telopeptide; BMI: Body Mass Index; TBFM: total body fat mass; TBLM: total body lean mass; TH: total hip; TBLH BMD: total body less head bone mineral density
